# Stability of the retinal image under normal viewing conditions and the implications for neural adaptation

**DOI:** 10.1038/s41598-024-52612-4

**Published:** 2024-01-27

**Authors:** Silvestre Manzanera, Pablo Artal

**Affiliations:** Laboratorio de Óptica, Universidad de Murcia, Campus de Espinardo (Edificio 34), 30100 Murcia, Spain

**Keywords:** Vision disorders, Applied optics, Visual system

## Abstract

Previous studies have demonstrated that the visual system adapts to the specific aberration pattern of an individual’s eye. Alterations to this pattern can lead to reduced visual performance, even when the Root Mean Square (RMS) of the wavefront error remains constant. However, it is well-established that ocular aberrations are dynamic and can change with factors such as pupil size and accommodation. This raises an intriguing question: can the neural system adapt to continuously changing aberration patterns? To address this question, we measured the ocular aberrations in four subjects under various natural viewing conditions, which included changes in accommodative state and pupil size. We subsequently computed the associated Point Spread Functions (PSFs). For each subject, we examined the stability in the orientation of the PSFs and analyzed the cross-correlation between different PSFs. These findings were then compared to the characteristics of a distribution featuring PSF shapes akin to random variations. Our results indicate that the changes observed in the PSFs are not substantial enough to produce a PSF shape distribution resembling random variations. This lends support to the notion that neural adaptation is indeed a viable mechanism even in response to continuously changing aberration patterns.

## Introduction

The human eye, responsible for forming images on the retina, does not constitute a perfect optical system. Consequently, when light converges onto the retina from a point-like object, the resulting wavefront is not spherical, and the image formed is not a perfect point. This phenomenon is influenced by optical aberrations. Lower-order aberrations, such as defocus and astigmatism, were first described in the nineteenth century^1^ and have traditionally been corrected using spectacles. In recent times, contact lenses and refractive surgery have also become viable options. Higher-order aberrations, beyond defocus and astigmatism, were first introduced by Helmholtz in 1881^2^ and later measured by Smirnov using a psychophysical method^3^. Since then, various techniques have been developed, including the spatially resolved refractometer^4,5^, laser ray tracing^6,7^, the double-pass method^8–10^, and the Hartmann-Shack (H–S) wavefront sensor^11,12^. The H–S sensor, in particular, has been extensively employed to measure ocular aberrations in large populations^13–16^, providing valuable statistics and revealing that each eye possesses a distinct and unique aberration pattern.

However, the performance of the visual system is not solely dependent on the optical quality of the eye. Cellular organization within the retina and neural post-processing also play crucial roles. Neural adaptation mechanisms have been observed in various aspects of the visual process, such as adaptation to chromatic aberrations^17^, image blur^18,19^, or the aberrations, primarily astigmatism, experienced by users of progressive power lenses^20^, who nonetheless adapt comfortably in most cases.

The notion that the visual system could adapt to the specific higher-order aberration pattern of an individual's eye was a hypothesis that required testing. However, the necessary experiment involved real-time manipulation of the wavefront, only made possible with the development of Adaptive Optics (AO). Initially designed for Astronomy to correct atmospheric turbulence-induced blur in telescope images^21^, AO was later successfully applied to correct human eye aberrations^22–24^ and simulate various optical conditions in an Adaptive Optics Visual Simulators (AOVS)^25–27^. By using an AO instrument, Artal and colleagues^28^ tested the adaptation hypothesis. Their experiment involved presenting subjects with a stimulus viewed through either their own aberrations or a rotated version of them. For all subjects, the stimulus appeared sharper when viewed through their own normal aberrations. This was the first substantial evidence supporting the adaptation hypothesis. Subsequent studies provided further corroboration^29–31^.

Certainly, this adaptation process would require a certain degree of stability in the ocular aberration pattern. Nevertheless, eye’s aberrations are known to be not entirely stable. They change gradually with age^32,33^, exhibit continuous fluctuations (2 Hz) of very short amplitude^34^, and are influenced by changes in pupil size and accommodative state^35,36^. Thus, is it possible for an adaptation process to occur under these changing conditions? Small fluctuations in amplitude or very slow changes related to aging likely would not pose significant obstacles. However, changes induced by pupil size and accommodation are more important and occur continually during normal visual activities.

This study aims to assess the extent to which changes in pupil size and accommodative state affect the stability of the ocular image quality. Moreover, it investigates how these changes impact the image on the retina and whether they are compatible with neural adaptation. To achieve this objective, we conducted measurements of ocular aberrations in four subjects under various natural viewing conditions, including variations in accommodative state and pupil size, and subsequently computed the associated Point Spread Functions (PSFs). We analyzed the stability of the different PSFs for each subject. Furthermore, to extend our analysis to a broader population, we repeated the same procedures for a large dataset of computationally generated ocular aberrations based on statistical data from previous studies involving large populations.

## Results

### Ocular point-spread functions (PSFs)

**Figure 1:**
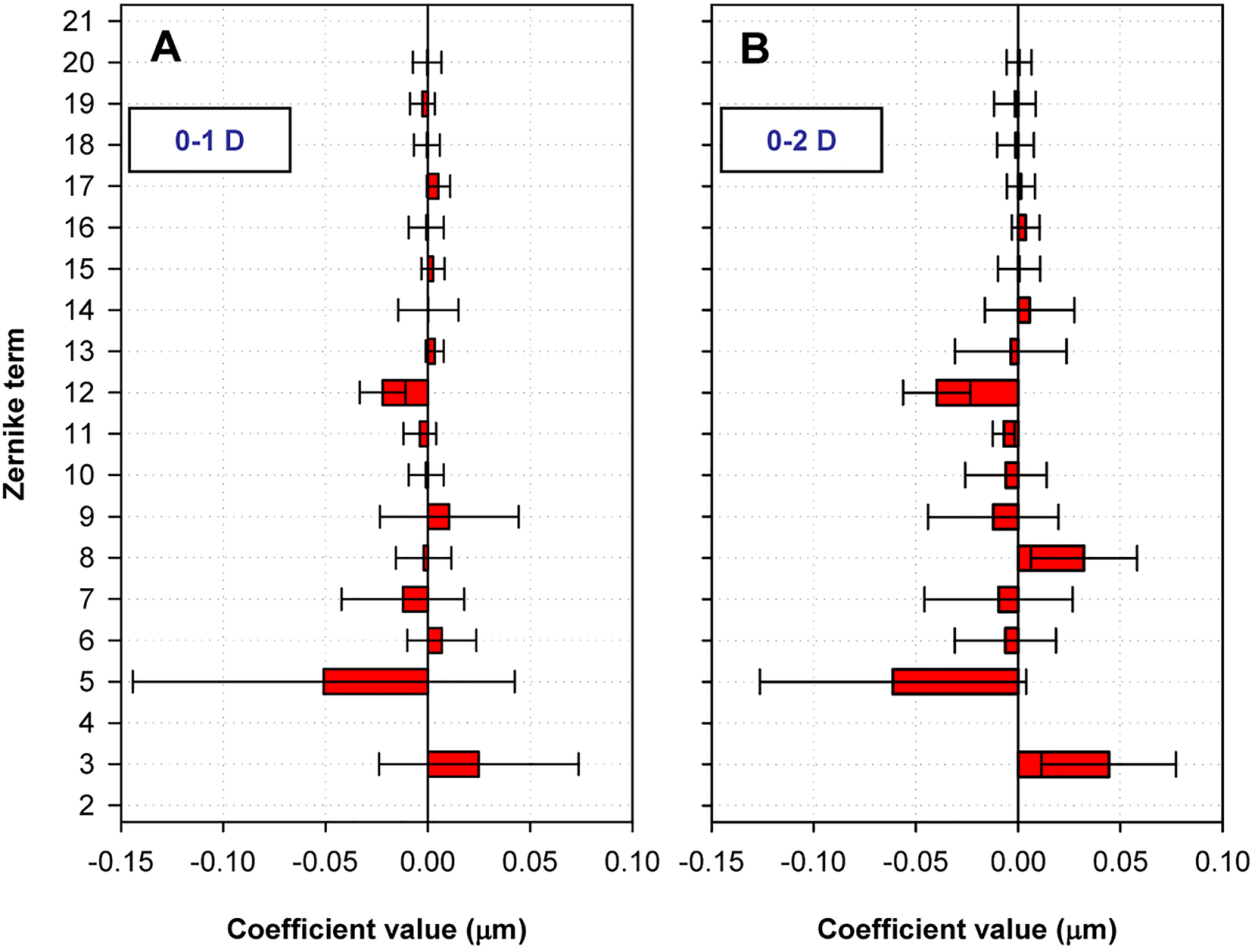
Through subjects average aberration change with accommodation at 5 mm pupil. Panel (**A**): changes from 0 to 1 D. Panel (**B**) : changes from 0 to 2 D. Changes in the 4th coefficient corresponding to defocus were deliberately set to zero.

To obtain the PSFs, wave aberrations were measured for subjects S1-S4 under various conditions, including 4, 5, and 6-mm pupil diameters and 0, 1, and 2 Diopters (D) of accommodation. To ensure data reliability, the average aberration changes with accommodation were initially calculated for a 5 mm pupil diameter. These results, as illustrated in Fig. 1, align well with previously reported findings in the literature. Notably, there is a shift toward negative values in spherical aberration (12th coefficient).

**Figure 2:**
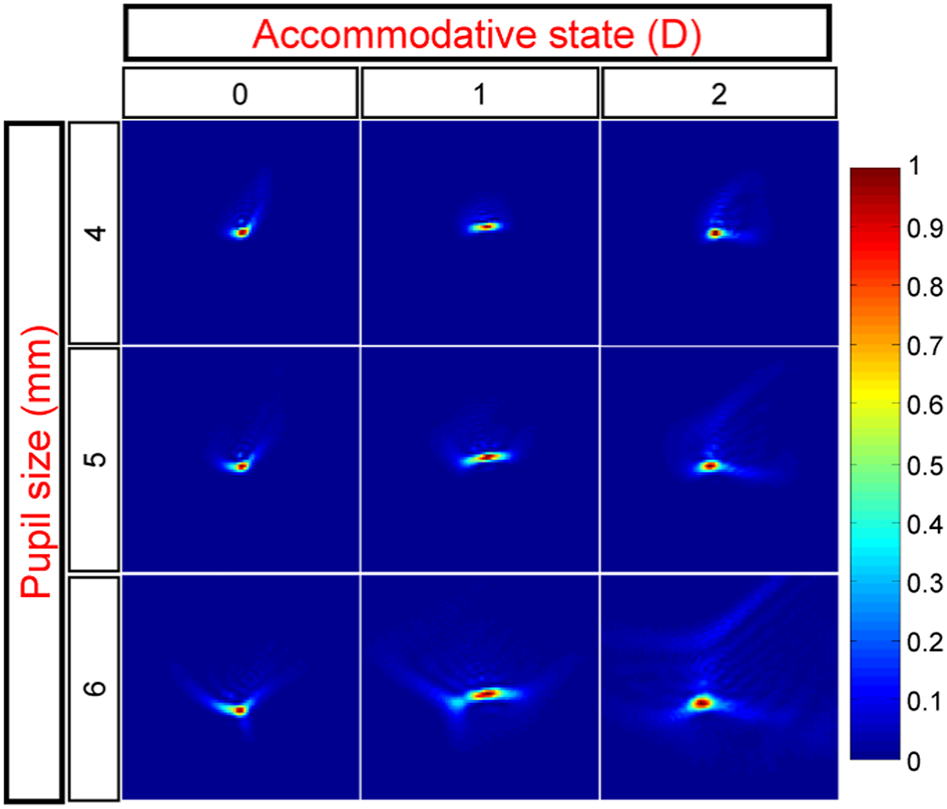
PSFs obtained for subject S2. The accommodative state and the pupil size at which each PSF was obtained are indicated by the corresponding column and row. To enhance details, each image is normalized to its maximum.

Subsequently, PSFs were computed based on the wavefront aberration measurements. For each subject, a 9-element matrix was generated, representing the PSFs for different accommodative states and pupil sizes. Figure 2, showcasing the PSFs for subject S2, serves as an illustrative example of the results obtained for the remaining subjects. As anticipated, smaller pupils result in more compact PSFs. Additionally, the impact of increased aberrations due to accommodation can be observed when comparing the PSFs at 2 D of accommodation with those at 0 D.

**Figure 3:**
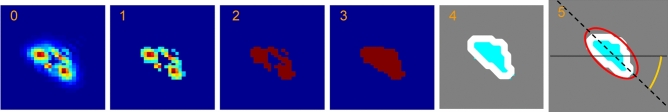
Example showing the procedure to obtain the orientation of a PSF. Each number at the upper left corner relates to the corresponding step number in the text. (0) original PSF, (1) thresholding, (2) binarizing, (3) filling, (4) boundaring, (5) angle.

These images were used to determine PSF orientation and cross-correlation and correspond to label 0 in Fig. 3. From the collection of virtual eyes previously generated, a similar 9-element PSF matrix was derived for each virtual aberration.

### PSFs’ orientation

Prior to calculating the orientation of the PSFs, a study was conducted to assess the degree of elongation in these PSFs. A circularly shaped PSF lacks a preferred orientation, rendering orientation analysis noisy and potentially meaningless. To address this, the eccentricity of the ellipse with the same second moments as the analyzed PSF (as described in the methods section) was computed for each PSF and then averaged for each subject. The results, 0.84, 0.9, 0.92, and 0.86 for subjects S1 to S4 respectively, demonstrate the existence of preferred orientations for all four subjects, thereby indicating the significance of estimating PSF orientations.

**Figure 4:**
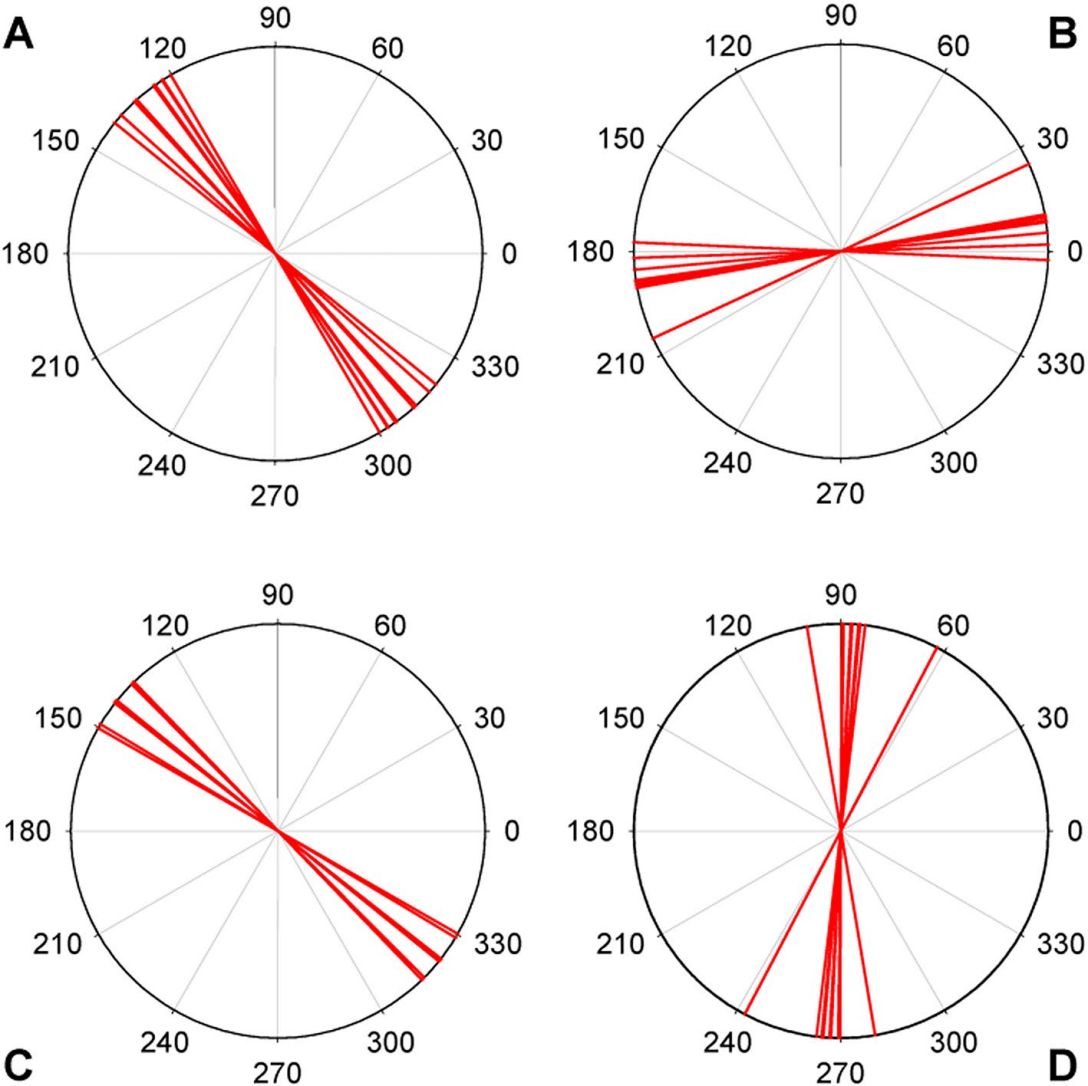
Diagrams showing the PSFs orientation for all the subjects. Panels (**A**), (**B**), (**C**) and (**D**) correspond to subjects S1, S2, S3 and S4 respectively. The orientation of each PSF corresponding to the three different pupil sizes and three different accommodative states is represented by each one of the 9 red lines inside each circle. Some lines are so close together that make it difficult to differentiate them.

Subsequently, these estimations were carried out, and the results are displayed in Fig. 4, providing an overview of the angular range within which PSF orientation changes with varying pupil size and accommodative state. For all four tested subjects, this range was relatively narrow, mostly below 30°, with a maximum of 37° (observed in subject S4). The next critical question to address is whether this variability in PSF orientations is sufficiently small to allow for an adaptation process. Answering this question, however, is challenging because our understanding of the prerequisites for such a process remains limited. On one hand, if the PSF orientation were consistently constant, we could assert that adaptation is possible. Conversely, if the orientation changed randomly, adaptation would not be feasible. Hence, comparing the observed changes in orientation with those in a hypothetical scenario of random PSFs would help determine the feasibility of the adaptation process. More precisely, it would enable us to decide whether these changes in PSF shape are definitively a limiting factor or not. To accomplish this, we computed the mean relative angle change for each PSF within each subject and then averaged these results across all subjects. This procedure was applied to both real and virtual subjects, yielding a comprehensive indicator of PSF stability. Notably, in the case of virtual eyes, the collection comprised 20,000 randomly generated eyes, all with a PSF eccentricity greater than 0.8 for the 0 D and 6 mm pupil case. As explained earlier, this criterion ensured the meaningful determination of changes in PSF orientation. This specific eccentricity value was chosen based on visual inspection of a sufficiently large sample of PSFs and was deemed reasonable. The sample size was determined^37^ to achieve a statistical power of 90%, enabling the detection of a difference on the order of 1 degree, with a significance threshold set at 0.05. This calculation takes into account an estimated standard deviation of approximately 30 degrees.

**Figure 5:**
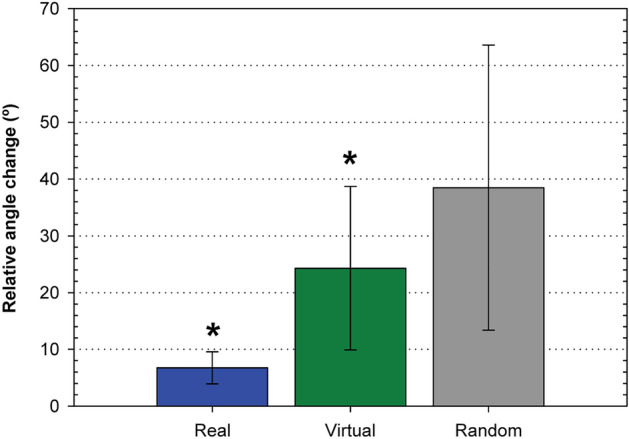
Relative angle change. Average for all subjects of the mean relative angle change for the different conditions of pupil size and accommodative state for the measured subjects and for the generated virtual subjects. They are compared with the average relative angle change of 20,000 randomly generated PSFs (see text for details). Error bars are ± 1 standard deviation. Asterisk indicates a statistically significant difference with the random case (*p* < 0.05).

To estimate how PSF orientations would change in a scenario where there is no relationship between them—i.e., where their relationship is random—we utilized the previously obtained collection of virtual eyes. For each PSF in this collection, we calculated the relative angle change with respect to the first generated PSF and then computed the mean change. Figure 5 provides a comparison of the mean relative angle changes for real subjects, virtual subjects, and random PSFs. A t-test was conducted, revealing that the difference in relative angle changes between real and virtual subjects compared to the random case is statistically significant (*p* < 0.05).

### PSFs’ cross-correlation analysis

**Figure 6:**
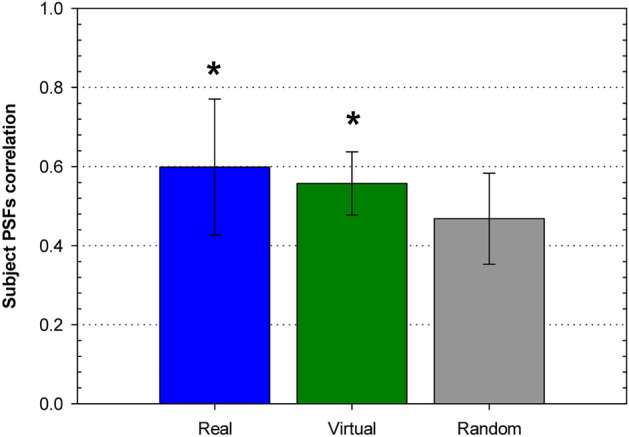
PSFs correlation. Average through subjects of the subject PSFs correlation parameter for the measured and virtual subjects. They are compared with the mean of the maximum values obtained from the cross-correlations performed over every pair of a 100 randomly generated PSFs collection (see text for details). Error bars are ± 1 standard deviation. Asterisk indicates that the difference with the random case is statistically significant (*p* < 0.05).

We have also conducted an analysis of PSF changes using the cross-correlation technique, as detailed in the method section. In line with the rationale applied in the previous orientation analysis, we compared the PSF changes observed in real and virtual subjects with those resulting from a scenario involving random PSFs. For both real and virtual subjects, we calculated the subject-specific PSF correlation parameter and subsequently computed the average across all subjects. The virtual subjects analyzed were part of a newly generated collection of 100 randomly generated virtual eyes. This number ensures a statistical power of 90% to find differences of 0.1 with an estimated standard deviation of 0.12 and a significance threshold of 0.05^37^. To determine the corresponding parameter in a situation where the relationship between PSFs is entirely random, we utilized the same collection of virtual eyes. In this case, we computed the cross-correlation between all possible pairs of PSFs and then calculated the average of the maximum correlation values. The results, presented in Fig. 6, illustrate the difference between the mean PSF correlation among subject PSFs in real and virtual eyes compared to the cross-correlation among random PSFs. These observed differences hold statistical significance (t-test, *p* < 0.05), thereby allowing us to assert that across changes in pupil size and accommodation, a certain degree of overall PSF shape is preserved.

## Discussion

We employed two distinct techniques to assess changes in the retinal Point Spread Function (PSF) resulting from alterations in ocular wavefront aberrations under varying conditions of accommodative state and pupil size. The analysis of PSF variability was conducted in two ways: first, by examining the stability in PSF orientation using image processing tools, and second, by evaluating the constancy of the overall PSF shape through cross-correlation analysis. When considering the aberrations measured in four subjects, both techniques concurred in demonstrating that, despite well-documented changes induced by accommodation and pupil size, these changes remain smaller than those anticipated under scenarios involving random variations in aberrations. This random scenario represents the limit of an adaptation process that cannot take place. This same conclusion holds for a large population of virtual eyes generated using existing knowledge about ocular aberration statistics and the impact of accommodation and pupil size. Consequently, we have established that changes in wavefront aberrations attributed to accommodation and pupil size are not a limiting factor for neural adaptation processes to occur.

However, it is worth noting that, for both techniques, the degree of PSF orientation and shape preservation is higher in real eyes compared to virtual eyes. This discrepancy could be attributed either to the fact that the group of four subjects studied may not be statistically representative, or, more plausibly, that the difference arises from the inclusion of low-order aberrations in the analysis. The four subjects measured exhibited astigmatism values of approximately a quarter diopter, which is a common residual astigmatism in individuals not requiring correction. Nevertheless, for all subjects, the mean Root Mean Square (RMS) due solely to astigmatism is of a similar magnitude to the mean RMS associated with higher-order aberrations, and approximately three times greater than the RMS of the mean aberration change at 2 D (for a 5 mm pupil). It is possible that these lower-order aberrations serve as a cushion, partially mitigating the effects of aberration changes.

It is important to note that astigmatism was not removed from the aberration data since one of the primary objectives of this study was to analyze real PSF changes occurring in the eye during normal visual tasks. Since none of the subjects was typically corrected for astigmatism, it was not set to zero. However, determining the typical level of defocus affecting the image on their retinas is a more complex matter. Numerous factors can influence this estimation, including whether they typically wear glasses or contact lenses, the quality of their correction, the duration of daily wear, voluntary or involuntary accommodation, and more. The approach we adopted, allowing subjects to determine their best subjective focus using the Badal optometer, was considered the most appropriate in terms of simplicity and proximity to real-life scenarios.

Upon reviewing Figs. 5 and 6, it becomes evident that the differences with the random mode are more pronounced in the orientation estimations than in the cross-correlation analysis. This discrepancy should not be surprising, as the latter employs a considerably more stringent parameter. Two PSFs may share the same orientation but exhibit distinct overall shapes, resulting in a lower cross-correlation.

## Materials and methods

### Instrument and procedure for recording experimental PSFs

Four subjects, labeled S1 through S4, aged between 27 and 40, participated in the study. S1 and S2 are myopic with prescriptions of − 1.5 D and − 2.75 D, respectively. S3 has a hyperopia of 0.75 D, while S4 is emmetropic. For all of them, the measured astigmatism was around a quarter diopter. All research protocols aligned with the Declaration of Helsinki guidelines. The University of Murcia’s review board approved the study, and written consent was procured from each participant.

**Figure 7:**
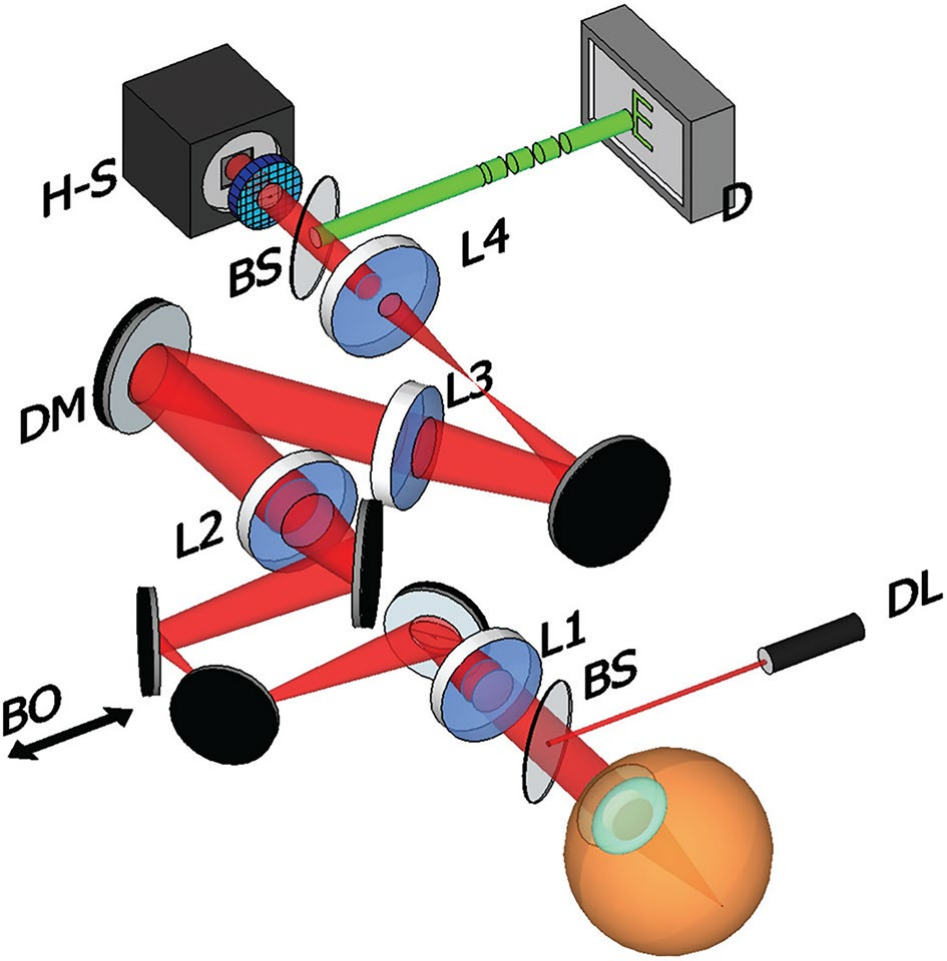
Diagram of the AOVS used in the experiment. A diode laser (DL) is used to illuminate the subject’s eye whose aberrations are measured by a Hartmann-Shack (H–S) wavefront sensor. A stimulus is shown on a distant display that the subject must keep in focus throughout the different vergences induced by the Badal optometer (BO).

The tool employed to estimate aberrations under varying conditions was an AOVS, with its schematic presented in Fig. 7. In this setup, a 780 nm wavelength infrared diode laser (DL) illuminates the eye after reflecting off a beam splitter (BS), establishing a beacon source on the retina. The lenses, L1 and L2, form a telescopic system that projects the eye's pupil plane onto the deformable mirror (DM) plane. In this experiment, the DM is flattened to function like a conventional mirror. Subsequently, another telescope, made up of lenses L3 and L4, projects the eye’s pupil onto the H–S wavefront sensor. A BS positioned before the lenslet array enables participants to view a stimulus on a distant display (D). The vergence of this stimulus can be adjusted by altering the gap between the two mirror pairs in the Badal optometer (BO). Aberration measurements took place under natural pupil conditions because a key objective was to assess the eye's aberrations across different accommodative states. Using mydriatics might have influenced the ciliary muscle's function. In the darkened room used for the measurements, all subjects had pupil sizes up to 6 mm. All tests were conducted without any corrective aids like glasses or contact lenses. After positioning the eye's pupil within the apparatus, the next step was to neutralize the spherical ametropia for every participant. Participants were allowed to adjust the BO to achieve the clearest possible image of the stimulus on the display. For reliable focus correction, this process was repeated thrice, and the average of the results was established as the final correction. This position served as the baseline for further accommodative demands on the participant. Three videos of 30 frames each were recorded at 25 frames/s at this focus position, capturing the spots from the H–S sensor. To mitigate noise impacts, frames within each video were averaged to produce one image, later used to determine wave aberrations at pupil sizes of 6, 5, and 4 mm, described as series of Zernike polynomials. Each pupil size's final wave aberration estimate is the average of the three sets of Zernike coefficients from each video. This process was replicated for 1 and 2 D accommodative states, with the stimulus vergence adjusted using the BO.

We acquired the ocular aberrations of each participant at pupil sizes of 4, 5, and 6 mm for 0, 1, and 2 D of accommodative states. These were then used to compute the monochromatic PSFs at the peak wavelength in the human spectral sensitivity curve using an FFT algorithm. It's worth noting that the fourth Zernike coefficient, which pertains to defocus, was adjusted for the eye's longitudinal chromatic aberration. To obtain the most accurate PSF, the aberrations, measured at 780 nm, were corrected by 0.8 D^38^. Other Zernike terms remain largely unaffected by wavelength^39–41^, hence no other corrections were needed.

### Determination of the virtual PSFs

In this study, we derived the PSFs of four actual subjects using real ocular aberration measurements. The data from these individuals, within an acceptable margin of error, can potentially generalize to the broader population. Moreover, by referencing previously published aberration statistics, we can gain further insights across a wider demographic. As previously discussed, the past decades have seen hundreds of eye measurements, with several studies detailing aberration statistics for typical eyes. A comprehensive review of these significant studies can be found in Salmon’s work^42^. Leveraging the mean and standard deviation of higher-order Zernike coefficients (up to the 5th order and 6 mm pupil) from this work, we generated a large number of statistically probable ocular aberrations. We achieved this by assuming a normal distribution probability for each coefficient. We use the term “virtual” to describe aspects linked to these statistically plausible yet non-real ocular aberrations.

Other studies have outlined the principal shifts in ocular aberrations under varying accommodative demands. One particular study^43^ presented findings for a general population with a 5 mm pupil. This research highlighted a linear relationship between changes in average spherical aberration and accommodation, with most other terms showing random changes that average out to zero. Using this data, we estimated aberration shifts for a 5 mm pupil during 1 and 2 D accommodative states, which were then extrapolated^44^ for a 6 mm pupil.

By merging the virtual aberration dataset with the anticipated changes due to accommodation, we now have three sets of Zernike coefficients for each virtual eye, representing aberrations at 0, 1, and 2 D. To factor in the effect of pupil size variations, we derived the wavefront from the Zernike coefficients and truncated it to pupil sizes of 5 and 4 mm. Consequently, we produced wave aberrations for 4, 5, and 6 mm pupil sizes at 0, 1, and 2 D of accommodative state for every virtual eye. Then, mirroring the procedure used for experimental PSFs, we employed an FFT algorithm to determine the monochromatic virtual PSFs.

For our virtual eyes dataset, the Zernike terms representing defocus and astigmatism were nullified. This is because the aberration statistics don't encompass these lower-order terms. Moreover, since these types of ametropia are frequently corrected in real life, incorporating them wouldn't offer an accurate representation of typical eye aberrations. Therefore, for our virtual eyes analysis, we focused solely on the stability of PSFs with only higher-order aberrations in consideration.

### Analysis of the PSFs

To assess the stability of the PSFs upon changes in accommodation and pupil size, we applied two methods: (i) analysis of the primary orientation stability of the PSF; (ii) cross-correlation between PSFs. Both methods were implemented using MATLAB© software. Further details on these methods are provided below.

Artal et al.’s 2004 study^28^ revealed that a rotation in the PSF can degrade overall visual performance. This implies potential adaptation to the unique shape or more specifically, the primary orientation of the PSF. Evaluating the stability of the PSF's orientation can thus provide insights into the feasibility of such adaptation. To determine the orientation of a given PSF from its raw image, the following procedure was adopted:

- Pixels with intensity values below 30% of the maximum PSF intensity were set to zero. This filters out low-intensity pixels that might skew orientation estimation. This 30% threshold was established after visually inspecting numerous PSFs.
- The PSF was binarized: pixels with non-zero intensity were set to 1. Objects with fewer than 2 pixels were eliminated, for reasons similar to step 1.
- The PSF was refined by filling gaps and smoothing edges and the boundaries of the PSF were then extracted.

The orientation of the PSF was identified as the angle between the horizontal axis and the major axis of an ellipse sharing the same second moments as the PSF region delineated. A visual representation of these steps can be found in Fig. 3. Instead of focusing on the specific angle of each PSF, we emphasized stability, or the relative angular changes as a function of accommodation and pupil size. To quantify this, we measured the absolute value of the difference between the angle of each PSF and a reference PSF (computed for 0 D and 6 mm pupil for each participant). This measure is henceforth termed the “relative angle change”.

To further evaluate PSF stability, the cross-correlation was performed between the PSFs as follows: for every participant, PSFs acquired under varying conditions of pupil size and accommodative state were thresholded using the previously mentioned 30% of maximum intensity as the threshold. Then all possible PSF pairs underwent normalized cross-correlation using the following expression^45^:

$$\gamma \left(u, v\right)= \frac{{\sum }_{xy}\left[f\left(x, y\right)- {\overline{f} }_{u,v}\right]\left[t\left(x-u, y-v\right)-\overline{t }\right]}{{\left\{{\sum }_{xy}{\left[f\left(x, y\right)- {\overline{f} }_{u,v}\right]}^{2}{\sum }_{xy}{\left[t\left(x-u, y-v\right)-\overline{t }\right]}^{2}\right\}}^{0.5}}$$

where $f$ and $t$ denote each of the correlated PSFs. The peak value from each correlation was determined, and these peak values were averaged to yield a single metric reflecting the overall similarity across a subject's PSFs. This metric is hereafter called the "subject PSFs correlation”.

## Data Availability

The datasets used and analyzed during the current study available from the corresponding author on reasonable request.
